# White-Matter Repair as a Novel Therapeutic Target for Early Adversity

**DOI:** 10.3389/fnins.2021.657693

**Published:** 2021-04-09

**Authors:** Rafiad Islam, Arie Kaffman

**Affiliations:** Department of Psychiatry, Yale University School of Medicine, New Haven, CT, United States

**Keywords:** early adversity, early life stress, myelin, white matter, animal models, translational research, rodents, non-human primates

## Abstract

Early adversity (**EA**) impairs myelin development in a manner that persists later in life across diverse mammalian species including humans, non-human primates, and rodents. These observations, coupled with the highly conserved nature of myelin development suggest that animal models can provide important insights into the molecular mechanisms by which EA impairs myelin development later in life and the impact of these changes on network connectivity, cognition, and behavior. However, this area of translational research has received relatively little attention and no comprehensive review is currently available to address these issues. This is particularly important given some recent mechanistic studies in rodents and the availability of new agents to increase myelination. The goals of this review are to highlight the need for additional pre-clinical work in this area and to provide specific examples that demonstrate the potential of this work to generate novel therapeutic interventions that are highly needed.

## Introduction

EA in the form of childhood neglect, poverty, and maltreatment is now recognized as one of leading causes for chronic mental and medical conditions in adulthood (Anda et al., 2006; Nemeroff, 2016; Teicher and Samson, 2016; White and Kaffman, 2019). Roughly half of all childhood psychiatric conditions are linked to EA (Green et al., 2010) and the cumulative economic burden of EA is estimated at $2 trillion in the United States alone (Peterson et al., 2018). Interventions that are able to correct maladaptive behaviors and cognitive deficits associated with EA are highly needed and will have tremendous clinical and economic impact. In this review we focus on the role that abnormal myelin development plays in mediating some of the psychiatric consequences of EA and propose that adaptive myelination may provide an important and novel therapeutic target for normalizing cognitive and behavioral deficits associated with EA.

Myelin is a vertebrate-unique evolutionary feature in which specialized glia cells in the central nervous system, known as oligodendrocytes, wrap myelin sheaths along axonal segments. This process starts at around birth and matures in early adulthood (Foster et al., 2019). Recent work from several groups has highlighted important new features of myelin development and its role in adult plasticity (Chorghay et al., 2018; Foster et al., 2019; Liu et al., 2020). During development, myelin facilitates the selection of functional connections and promotes circuit synchronization and oscillatory outputs (Pajevic et al., 2014; Chorghay et al., 2018; Foster et al., 2019; Liu et al., 2020). This process is tightly linked to neuronal activation and is sensitive to environmental changes early in life (Foster et al., 2019; Liu et al., 2020). Not surprisingly, exposure to EA in the form of neglect, deprivation and maltreatment alters white-matter development in humans, non-human primates and rodents (reviewed in Table 1). Although some progress has been made in identifying the mechanisms by which EA alters myelin development, the findings are inconsistent and many key questions have not been addressed. Theses inconsistencies are likely due to the heterogeneity in the type, timing, and complexity of the adversity, as well as genetic vulnerability and brain region-specific changes in activity-dependent myelination (aka ***adaptive myelination***).

**TABLE 1:** EA impairs myelin development in humans, non-human primates and rodents.

**References**	**Experimental design**	**Summary of findings**
**Humans**		
Teicher and Samson, 2016	Systematic review	Reduction in the size of the corpus callosum and reduced fractional anisotropy in the corpus callosum as the most robust findings in humans exposed to EA. Also, summarizes findings in other white matter tracks including the inferior longitudinal fasciculus, superior longitudinal fasciculus/arcuate fasciculus, uncinated fasciculus, cingulum bundle and the fornix
McCarthy-Jones et al., 2018	Diffusion MRIs were used to assessed the effects of EA on microstructural integrity (i.e., fractional anisotropy, tissue fractional anisotropy and free water diffusion) in 7 white tracks that have previously been shown to be altered by childhood adversity. EA was assessed using Childhood adversity questionnaire (no childhood adversity *n* = 34, low score of EA *n* = 58, high score of EA *n* = 55, ages 37–42). ROI included cingulum bundle, corona radiata, corpus callosum, superior longitudinal fasciculus, inferior frontal-occipital fasciculus, fornix and the uncinate fasciculus.	Found significant reduction in FA in 6 out of the 7 ROI when using EA as a categorical variable, with the fornix being the only ROI that was not significant using categorical analysis. When EA was used as a continuous variable all 7 ROI showed significant changes in FA using regression analysis.
Bick et al., 2015	Randomized clinical trial examining the effects of adoption into foster families on white matter development in children that were institutionalized in a Romanian orphanage during the first 2 years of life and then either stayed in the institution (IG) or adopted (AG). Institutionalized group (IG) *n* = 26, adopted group (AG) *n* = 23, never institutionalized group (CTL) *n* = 20, ages 8–11 when imaged.	Normalization of FA were seen in the AG but not the IG groups in several white tracks including the left external capsule, right external capsule, retro-lenticular internal capsule, right cingulum, right anterior corona radiata, left superior corona radiata, and the medial lemniscus. No remediation of foster care were seen for the corpus callosum and the superior corona radiatum.
Tanti et al., 2018	Postmortem assessment of markers of myelination in the ventro-medial prefrontal white matter region in individuals that died by suicide in the context of depression and EA (DS-EA), depression but without EA (DS) and no depression and no EA, CTL (*n* = 18 males per group, average age 37)	Reduced density of Olig2 (a general marker of OL) and increased density of Nogo-A, APC, Mash1 (markers of mature OL) in DS-EA compared to DS and CTL groups with some evidence that these effects were more pronounced in younger individuals. DS-EA and DS groups show reduced MBP levels but there was no effect of EA on any markers of myelination (e.g., PLP, CNP, MAG, MOG, MOBP). The authors propose that EA causes precocious maturation of OPC in the prefrontal cortex.
**Non-human primates**		
Sanchez et al., 1998	MRI changes were assessed in rhesus monkeys (*Macaca mulatta*) exposed to nursery (singly caged, 2–6 months or 2–12 months) or semi-naturally social rearing, from 2 to 12 months, with behavioral testing done at 12M and MRI at 18M (Nursery, *n* = 9 males, Control, *n* = 11 males)	Single caging caused a reduction in corpus callosum size, mainly in posterior/caudal regions. Reduced corpus callosum size persisted after 6 months of peer rearing and was correlated with slower acquisition, but not performance, in the delayed non-matching to sample task. Reduced white matter volumes were also seen in the prefrontal cortex and the parietal cortex.
Coe et al., 2002	Pregnant female rhesus monkeys (*Macaca mulatta*) were exposed to daily 10 min acoustic startle during the third trimester (gestational ages 90–140). After birth, infants were raised in nursery for 1 month and then in a small peer-group. MRIs were done at 7–11 months of age. CTL *n* = 5, Prenatal stress *n* = 11, roughly 50% males.	There was a significant interaction between prenatal stress and sex for corpus callosum size with reduced volumes in males and increased volume in females. These changes were most pronounced in posterior/caudal regions of the corpus callosum.
Jackowski et al., 2011	Mother-infant dyads of Bonnet monkeys (Macaca radiata) were exposed to variable foraging demands (VFD) when infants were 2–6 months. In adulthood male monkeys were scanned with MRI (CTL *n* = 9, VFD *n* = 14, roughly 5 year old. Two years later some of the animals were tested in the intruder test and processed for postmortem assessment of corpus callosum mid-sagittal surface area.	Exposure to VFD early in life was associated with reduced corpus callosum size using a region of interest, but not whole brain voxel volumetric assessment. Reduced corpus callosum was highly correlated with increased emotional reactivity in the intruder test and was confirmed using postmortem analysis performed 2 year after the initial MRI study. Together these findings indicate that exposure to VFD causes long-term reduction in corpus callosum size.
Howell et al., 2013	A naturalistic childhood maltreatment model in rhesus monkeys (*Macaca mulatta*) was used to assess the effects of childhood maltreatment on white matter organization (FA, RD, AD) using dMRI in adolescent animals. CTL *n* = 10 (6F/4M), MT *n* = 9 (5F/4M), ages around 4 years.	Maternal maltreatment was associated with reduced FA in the corpus callosum and several other white- matter tracks including the occipital white matter and brain stem. Reduction in FA was correlated with elevated corticosterone levels at 1 month of age when maltreatment was at its peak. Reduced FA in occipital WM was correlated with increased aggression in adolescence.
**Rodents**		
Makinodan et al., 2012	Tested the effects of social isolation during the juvenile period (P21-55) on social behavior, PFC function (non-match to place task) and myelination in mice.	Social deprivation during a sensitive period of development (P21-P35) causes abnormal maturation of oligodendrocytes in the PFC. Abnormal myelination in the PFC is due to impaired Erbb3 receptor activation in oligodendrocytes leading to social and PFC deficits that are not easily reversed later in life.
Yang et al., 2017	Sprague Dawley rats were exposed to a split-litter maternal separation model, in which half of the pups were isolated from littermates and dam for 3 h from P3-21. The remaining pups were handled but immediately returned to the home-cage and served as controls. Pups were weaned at P21 and assessed for myelination at P21 or as young adults (P60). The exact timing for the behavioral work was not specified, except for those rats tested after administering the WNT antagonist XAV939.	MS impaired myelin formation in the PFC, but not in other brain regions such as the corpus callosum. Deficits in myelination were seen in P21 pups and persisted into adulthood. The authors provide compelling evidence that increased WNT signaling prevents the maturation of oligodendrocytes in the PFC of rats exposed to MS, including data showing that blocking WNT signaling can rescue behavioral and myelination deficits seen in MS rats. They further show that demyelinating injury to the PFC causes similar behavioral abnormalities to those seen in rats exposed to MS.
Teissier et al., 2020	Balb/cj mouse pups were exposed to 3 h of MS from P2-14. Effects of MS and neuronal activation/inactivation on myelination were tested at P15 and adulthood (P80) in the PFC. Effects on behavior were tested in adulthood.	MS caused reduced expression of immediate early activation genes in the PFC (i.e., reduced neuronal activation) that was associated with accelerated maturation of OPC into mature OL at P15, and reduced OPC density, but not mature OL in the PFC of adult mice. Using chemogenetic manipulation of neurons in the PFC the authors showed that inactivation of PFC neurons from P2-14 in mice raised under CTL condition is sufficient to recapitulate the myelination and behavioral deficits seen in MS mice. Further, activation of neurons in the PFC during the first two weeks of life eliminated the behavioral abnormalities seen in MS mice.
Berrebi et al., 1988	Rat pups were separated from their dam and littermates for 3 min from P1-20 (i.e., handled) and corpus callosum size was assessed at P110 and P225.	Handling increased corpus callosum size in P110 (fully adult rats) males and reduced it in females. The interaction between sex and rearing for corpus callosum size was no longer significant at P215 (middle-age rats).
Duque et al., 2012	Used maternal separation with early weaning (MSEW) in which C57BL/6j pups were exposed to prolonged maternal separation (4–8 h) from P2-P17 and weaned at P17. Control (CTL) pups were left undisturbed and weaned at P23. Stereological and DTI measurements were done in adult male mice (P75-95).	MSEW adult mice showed reduced total brain volumes that was most pronounced in the left hemisphere and was particularly notable in the cortex and the hippocampus. Reduced gray matter volume were associated with abnormal myelination in several white matter tracks including reduced myelination and FA in the rostral and medial regions of the corpus callosum. Again, these were more pronounced in the left hemisphere and were associated with increased Olig2 density in the left CC and PFC of MSEW male mice.
White et al., 2020	Balbc/Byj mice pups were exposed to unpredictable postnatal stress (UPS). UPS includes raising pups with limited bedding and nesting from P0-25 and exposing them to unpredictable 1 h maternal separation/nest disruption on P14, P16, P17, P21, P23, P25. In adulthood (P75-90) mice were processed for high resolution dMRI (6 mice per rearing condition and sex, for a total of 24 mice)	Whole brain voxel analysis found reduced volume and FA in the rostral regions of the corpus callosum in UPS mice (cluster size > 25, FDR < 0.1). These changes were seen in both hemispheres with similar outcomes in males and females. UPS mice also showed reduced global network efficiency and increased in small-woldness.

*AD, axial diffusivity; AG, adopted group; dMRI, diffusion MRI; EA, Early adversity; FA, Fractional anisotropy; FDR, false discovery rate; IG, institutionalized group; MS, Maternal separation; MSEW, maternal separation with early weaning; OL, Oligodendrocytes; OPC, Oligodendrocytes progenitor cells; P21, postnatal day 21; PFC, prefrontal cortex; RD, radial diffusivity; ROI, region of interest; UPS, unpredictable postnatal stress; VFD, variable foraging demands.*

The central assertion of this review is that animal models can untangle many of these challenges and clarify how different types of adversities impair myelination in a manner that persists into adulthood and impacts cognition and behavior. Further, we note that this area of translational research has received relatively little attention and call for additional studies. In support of this assertion we note that myelin development is highly conserved across diverse mammalian species (Bergles and Richardson, 2015). Key variables of adversity can be rigorously controlled in animals and sophisticated tools are available to manipulate neurons and oligodendrocytes in animals. Further, the availability of human imaging techniques to assess myelination in animals allows for more direct cross-species comparisons (Kaffman et al., 2019), and recent progress in the development of therapeutic interventions that enhance adaptive myelination (Murphy and Franklin, 2017) provides a novel strategy to reverse myelin and behavioral abnormalities seen in animals exposed to EA.

We start with a general review of myelin function in nerve conduction, network synchronization, behavior, and summarize the role that oligodendrocytes play in network selection during development. Next, we review a large body of work showing that EA alters myelin development in humans, non-human primates and rodents, highlighting important mechanisms that appear to guide these changes in rodents. We close by outlining key unresolved questions and the work needed to address these issues.

## Myelin Modulates Network Properties in Response to Environmental Cues

**FIGURE 1:**
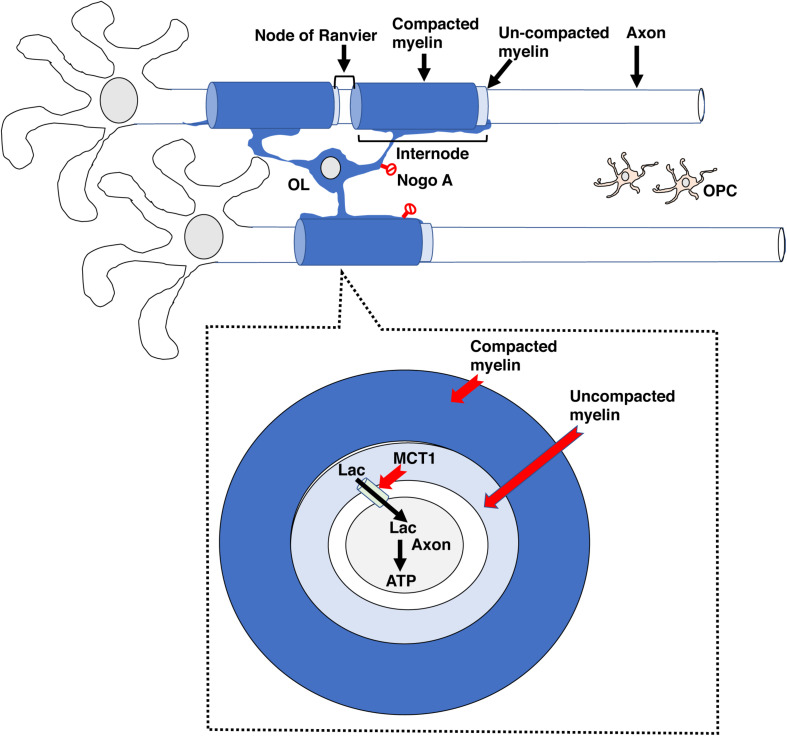
The basic myelin components. A single oligodendrocyte can myelinate several neighboring axons by forming internodal myelin sheaths that are interspaced with myelin free zones known as nodes of Ranvier. Compacted myelin (dark blue) increases nerve conduction and prevents axonal sprouting by expressing ligands such as Nogo A (no entry red signs), whereas uncompacted myelin (light blue) provides metabolic support to underlying axonal segment by expressing channels such as MCT1. Abbreviations: OL- mature oligodendrocyte, OPC- oligodendrocyte progenitor cell.

Oligodendrocytes (**OL**) are specialized cells that wrap lipid-rich myelin sheaths, known as ***internodes***, around large-diameter axons. Internodes with different lengths are spaced along axons at variable densities and are interspaced by specialized myelin-free segments enriched with ion channels known as ***nodes of Ranvier*** (Figure 1) and reviewed in Chorghay et al. (2018), Foster et al. (2019). Internodes prevent current leakage along large axonal segments leading to saltatory conduction with increased conduction velocity and greater reliability in signal transduction (Mitew et al., 2014; Chorghay et al., 2018). Conduction velocity is modulated by myelin thickness and internode length and density along the axon. These properties are highly regulated by axonal activity and environmental cues, a process known as ***adaptive myelination*** (Gibson et al., 2014; Foster et al., 2019; Suminaite et al., 2019). Adaptive myelination promotes network synchronization, the selection of functional circuits during development, improved learning, and behavioral modifications later in life (Pajevic et al., 2014; Kaller et al., 2017; Chorghay et al., 2018). See Sections “Myelin and Behavior,” “Mechanisms of Myelin Development,” and “EA Causes White-Matter Abnormalities in Humans, Non-human Primates and Rodents” below for more details.

Myelin also plays an important role in addressing energetic demands of neuronal networks (Saab et al., 2013; Philips and Rothstein, 2017). For example, insulation of large axonal segments minimize current leakage and reduces energetic costs associated with neuronal impulse propagation (Mitew et al., 2014). Neuros have small reserves of glycogen storage and therefore rely on metabolic support provided by astrocytes and oligodendrocytes, with oligodendrocytes playing a particularly important role in transporting lactate into adjacent axons Saab et al. (2013), Philips and Rothstein (2017). Monocarboxylate transporters such as MCT1 located in ***uncompacted myelin*** allows for the transport of lactate into the adjacent axon where it is converted to ATP (Figure 1). This metabolic coupling is highly regulated by axonal activity and is essential for its function, for reviews see Saab et al. (2013); Philips and Rothstein (2017). The importance of this “symbiotic” relationship is highlighted by work showing that oligodendrocyte-specific deletion of MCT1 causes axonal degeneration in aged mice (Lee et al., 2012; Philips et al., 2021). Perturbation in components of ***compacted myelin***, such as myelin basic protein, which are responsible increasing conduction velocity do not cause axonal degeneration, indicating that compacted and uncompacted myelin have different roles. This distinction may explain why some myelin changes associated with EA are more difficult to reverse later in life and why apparently similar perturbation in myelin structure may cause different functional consequences (Bick et al., 2015; Philips and Rothstein, 2017; McCarthy-Jones et al., 2018; De Leon Reyes et al., 2020).

Myelin also inhibits neuronal plasticity. This is mediated by the expression of ligands such as Nogo-A and MAG by oligodendrocytes (Figure 1, red no entry signs). These ligands restrict axonal sprouting and prevent axonal regeneration after spinal cord injury, stroke, and traumatic brain injury (Geoffroy and Zheng, 2014). Increased levels of myelination in the visual cortex during the juvenile period is responsible for closing the critical period in response to monocular deprivation (McGee et al., 2005). The ability of myelin to promote plasticity in activated axons while inhibiting sprouting in neighboring axons is a unique feature that may promote the selection of functional circuits during development and later in life.

In summary, myelin increases processing speed and accuracy at significantly reduced energetic costs. Myelin also provides metabolic support, helps synchronize network oscillation and restricts axonal sprouting. These properties are influenced by environmental cues and are necessary for selecting functional circuits and to improve learning and perception throughout life.

## Myelin and Behavior

Rapid changes in myelination are seen in response to different forms of learning, social cues, and stress, with a growing body of work suggesting that this form of myelin plasticity plays an important role in modifying circuit output, cognition, and behavior (Xiao et al., 2016; Kaller et al., 2017; Chorghay et al., 2018; Liu et al., 2020). For example, elegant work by McKenzie et al. showed that training mice to run on a wheel with missing rungs (aka complex running wheel) induces the differentiation of OPC into mature OL in the corpus callosum and that blocking this process impairs the acquisition but has no effect on the retention of this task after it was acquired (McKenzie et al., 2014). Increase in OPC differentiation is seen within 2.5 h of exposure to complex wheel running and this rapid response was required for maximize acquisition of this skill within this time frame (Xiao et al., 2016). Together these findings demonstrate rapid OPC differentiation and *de novo* myelination improve performance in complex procedural tasks.

Steadman et al. have extended these findings to show that *de novo* myelination is necessary for the consolidation/retrieval of spatial memory in the Morris water maze and contextual fear conditioning (Steadman et al., 2020). Consolidation of spatial memory was associated with increased synchronization between the hippocampus and the anterior cingulate of the prefrontal cortex and this enhanced synchronization was blocked when *de novo* myelination was prevented. Together, these findings establish an important link between *de novo* myelination, network synchronization, and spatial learning (Steadman et al., 2020).

**FIGURE 2:**
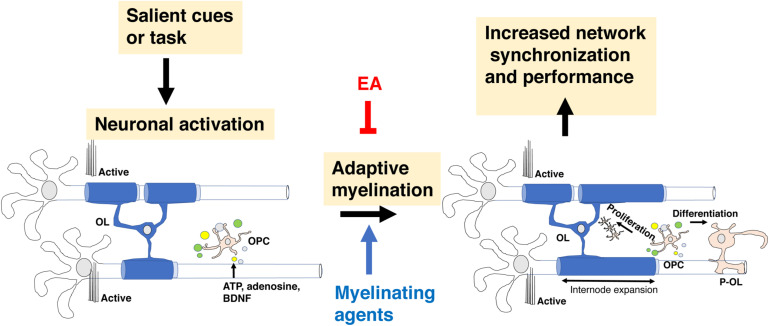
Adaptive myelination in adulthood. Environmental cues increase neuronal activity in specific circuits, leading to the release of ligands such as glutamate, adenosine and BDNF (small colored circles) that promote the expansion of existing internodes. In adult rodents, these ligands drive OPC proliferation and differentiation but this aspect of *de novo* myelination seems negligible in healthy adult humans. Adaptive myelination in adulthood increases network synchronization and improves performance in a variety of tasks. Myelinating agents such as Clemastine fumarate increase adaptive myelination while some forms of EA or prolonged social deprivation in adulthood impair it. EA, early adversity; OL, mature oligodendrocyte; OPC, oligodendrocyte progenitor cell.

Prolong social isolation reduced myelination in the PFC and was associated with a decreased social interaction in adult mice (Liu et al., 2012). Interestingly, administration of the anti-muscarinic agent Clemastine fumarate increased myelination in the PFC and normalized social avoidance in socially isolated mice (Liu et al., 2016). These observations suggest that social interaction is necessary for maintaining adequate levels of *de novo* myelination in the PFC and demonstrate the therapeutic utility of agents that increase *de novo* myelination for restoring normal social behavior (Figure 2).

Pan et al. (2020) showed that contextual fear conditioning was associated with a time- dependent increase in *de novo* myelination in the PFC. Increased myelination was seen roughly 14 days after fear learning and was not seen in the hippocampus or the amygdala. This time-dependent myelination was required for remote (i.e., 28-day after training) but not acute learning (i.e., 24 h post-training). Calcium imaging showed significant changes in neuronal activation in response to remote learning that required *de novo* myelination. Administration of Clemastine fumarate increased remote fear learning in a manner that required *de novo* myelination (Pan et al., 2020), further demonstrating the ability of these agents to modify complex behavior in adulthood.

In summary, *de novo* myelination plays a critical role in different forms of learning including procedural, spatial, and contextual fear conditioning. Myelin-dependent changes in behavior are linked to changes in synchronization and reorganization of neuronal network in specific brain regions, most notably the PFC. Importantly, agents that increase *de novo* myelination promote learning and can be used to compensate for behavioral deficits associated with environmental deprivation (Figure 2).

## Mechanisms of Myelin Development

**FIGURE 3:**
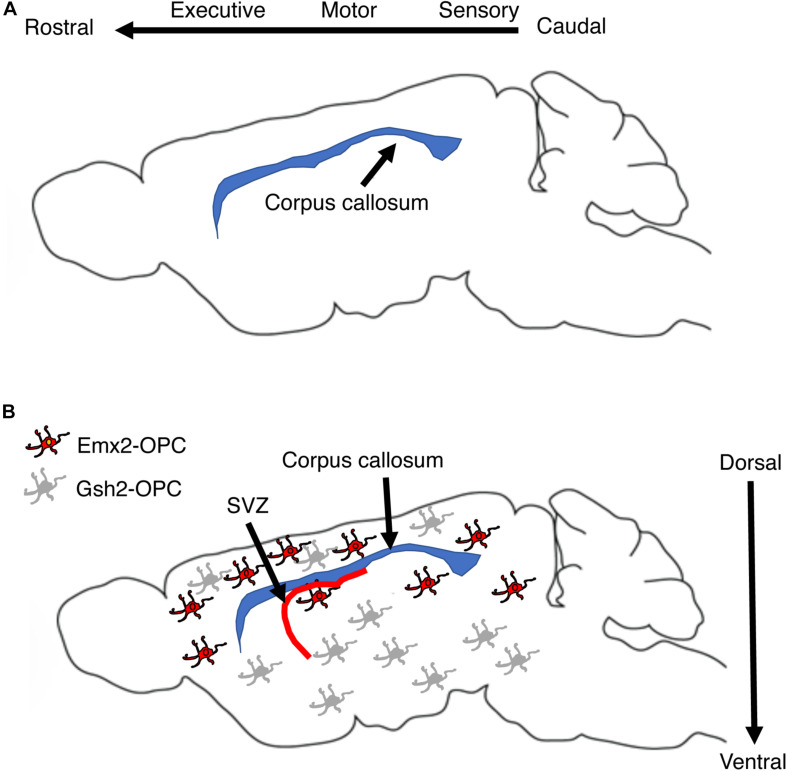
Corpus callosum and myelin development. **(A)** Myelination in the corpus callosum proceeds from caudal to rostral to ensure sequential maturation of circuits that enhance attachment and escape behavior early in life. **(B)** OPCs are a large, heterogenous and somewhat redundant cell population that undergoes significant changes in composition during the first 10 days after birth. Emx2-OPC (red) populate the dorsal telencephalon, appear to be mammalian specific, and are highly responsive to demyelinating conditions. Gsh2-OPC (gray) are present in both the dorsal and ventral telencephalon. Both Emx-2 and Gsh2 OPC continue to be generated throughout life from the SVZ (red). OPCs, oligodendrocyte progenitor cells.

Myelination during development is a protracted and highly coordinated process, starting at around birth and continuing into early adulthood. During development, myelination progresses in a caudal to rostral fashion that corresponds with maturation of sensory-motor, language, cognition, and executive functions respectively, Figure 3A (Mitew et al., 2014; Kaller et al., 2017; De Leon Reyes et al., 2020). Although adaptive myelination in adulthood and developmental myelination share some similarities, important differences exist that are particularly relevant to questions about long-term impact reversibility. Here we summarize key features of myelin development during the postnatal period and highlight their relevance to abnormalities seen in different forms of EA and for therapeutic interventions.

High degree of redundancy ensures robust myelin development under diverse environmental conditions or genetic perturbations (Mitew et al., 2014; Bechler et al., 2018). This redundancy has made it difficult to identify specific pathways that are indispensable for myelin development in the CNS (Mitew et al., 2014; Bechler et al., 2018) and suggest that the effects of EA on myelin development are likely to be multi-factorial and perhaps not simple to correct.

Multipotent neural stem cells differentiate into self-replicating oligodendrocyte progenitor cells (**OPC**), a process regulated by soluble factors such as sonic hedgehog, WNT/β-catenin, and bone morphogenic proteins (Ortega et al., 2013; El Waly et al., 2014; Mitew et al., 2014; Sanchez and Armstrong, 2018). Three waves of OPC migrate and populate the developing brain (Kessaris et al., 2006; Bergles and Richardson, 2015). The first wave is composed of Nkx2.1 positive cells that originates from the ventral neuroepithelium and populates the telencephalon by E16.5. A second wave of Gsh2-positive OPC originates from neighboring sections of the ventral neuroepithelium and arrives to the forebrain soon after the first wave. These embryonic waves are followed by a distinct third wave of Emx2-positive cells. This third wave is generated after birth within the cortex and populates only dorsal regions of the telencephalon such as the cortex and the corpus callosum. In the following 10 days after birth, the Nkx2.1 cell population is eliminated while the number of Emx2-positive cells in the dorsal telencephalon increases to levels that are similar to those of Gsh2-positive OPC. These changes are driven by the production of large numbers of Emx2 and Gsh2, but not Nkx2.1 OPC from the subventricular zone during the first two weeks of life, a process that continues albeit at a lower rate later in life, Figure 3B (Ortega et al., 2013; El Waly et al., 2014; Sanchez and Armstrong, 2018). Ablating Gsh-2 OPC leads to expansion of Emx2 population and vice versa, suggesting that heterogenous and somewhat redundant population of OPC competes for space in the developing telencephalon (Richardson et al., 2006). The observations that Emx2-positive OPC are restricted to dorsal areas and that equivalent wave of dorsally generated cortical OPC is not seen in birds led Richardson and colleagues to propose that these cells represent an important evolutionary step in mammalian cortical expansion (Richardson et al., 2006; Bergles and Richardson, 2015). Emx2, but not Gsh2 positive cells proliferate in response to demyelinating injury suggesting that despite significant overlap and redundancy these populations are not identical and are likely make distinct contribution for adaptive myelination and behavior later in life (Crawford et al., 2016). Further, differences in OPC composition between Gsh2 ventrally and Gsh2/Emx2 dorsally (Kessaris et al., 2006) might give rise to subtle differences in myelination along the dorsal-ventral axis (Bechler et al., 2018). The dynamic changes in OPC composition during the perinatal period make these processes good candidates for environmental perturbations including EA (Figure 3B). However, to the best of our knowledge the effects of EA on OPC composition during the postnatal period has not been investigated yet.

**FIGURE 4:**
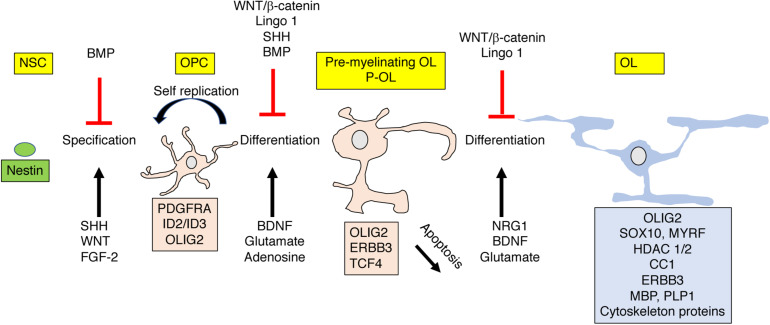
OPC differentiation and apoptosis are highly regulated by external signals. Nestin positive polypotent NSC differentiate into lineage specific OPC that give rise to pre-myelinating (P-OL) or undergo apoptosis. P-OL then differentiate into mature OL. These processes are tightly controlled by diffusible and non-diffusible molecules expressed by active or non-active axons. These instructive molecules can promote (black ↑) or inhibit (red ⊥) these processes by altering gene expression in these cells (shown as a box with matching color). Genes and proteins shown represent only a partial list of key molecules discussed in the text. For more comprehensive reviews see Mitew et al. (2014), Foster et al. (2019). NSC, polypotent neural stem cell; OPC, oligodendrocyte progenitor cells; P-OL, pre-myelinating oligodendrocytes; OL, mature oligodendrocytes.

Strict developmental cues instruct OPC to exit the cell cycle and differentiate into short-lived pre-myelinating oligodendrocytes that give rise to mature myelin producing oligodendrocytes (**OL**) or undergo apoptosis (Figure 4). Roughly 20–50% of the pre-myelinating oligodendrocytes undergo apoptosis during development, a decision that is tightly linked with their ability to contact and myelinate active axons (Barres et al., 1992; Trapp et al., 1997; Mitew et al., 2018). The rapid cell cycle and high levels of OPC apoptosis are unique features of developmental myelination and are order of magnitude lower in adult rodents (Barres et al., 1992; Mitew et al., 2018) and practically negligible in adult humans (Yeung et al., 2014). The high rate of OPC apoptosis during development appears to be driven by a competition for limited survival cues that ensure selective myelination of active axons (Barres et al., 1992; Mitew et al., 2018).

Signals such as Neuregulin 1 (NRG1), adenosine, glutamate, and BDNF, expressed and secreted from activated axons play a critical role in this differentiation process, Figure 4 (Stevens et al., 2002; Makinodan et al., 2012; Gibson et al., 2014; Wake et al., 2015; Mitew et al., 2018; Geraghty et al., 2019). The tight link between neuronal activation and OPC differentiation makes this process highly sensitive to perturbations induced by EA. For example, Makinodan et al. (2012) has shown that reduced Neuregulin 1-Erbb3 signaling is responsible for abnormal myelination in the PFC in socially deprived juvenile mice and Teissier et al. (2020) has established a causal link between neuronal activity and OPC maturation in a rodent model of EA (Table 1).

**FIGURE 5:**
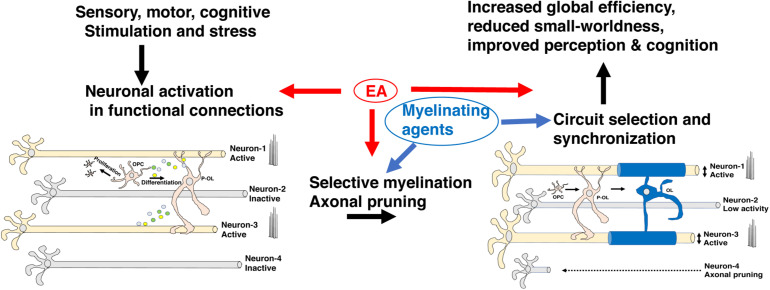
Myelin promotes the selection of functional circuits during development. Environmental stimulation or deprivation early in life leads to changes in neuronal activity in the developing brain. Increased activation in appropriately connected functional units (neurons 1 & 3) but not in neurons 2 & 4 leads to preferential myelination, enhanced metabolic support, and axonal growth in neurons 1 & 3 at the expense of neuron 2. Neuron 4 undergoes axonal pruning commonly seen during a critical period of transcallosal-projection development and is irreversible later in life. Adaptive myelination coupled with axonal pruning promotes synchronization of functional circuits and maturation of network properties (e.g., increased global efficiency and reduced small-worldness) that improve cognition and perceptual accuracy. Exposure to EA interferes with different aspects of this process and can potentially be reversed by myelinating agents. EA, early adversity; OL, mature oligodendrocyte; OPC, oligodendrocyte progenitor cell; P-OL, pre-myelinating oligodendrocytes.

During development, the brain wires itself by first casting an exuberant rudimentary network and then selecting functional connections and actively eliminating non-functional redundant connections (De Leon Reyes et al., 2020; Pan and Monje, 2020). This process of elimination takes place during a specific period of development, known as the critical period, and is difficult to reverse later in life (McGee et al., 2005; Kaffman and Meaney, 2007; Pan and Monje, 2020). Although still somewhat speculative, a growing body of work has suggested that myelination plays a critical role in promoting circuit refinement during critical or sensitive periods of development. For example, myelin’s ability to increase conduction velocity and enhance metabolic support in activated axons is likely to promote the retention of myelinated circuits during development (Saab et al., 2013; Chorghay et al., 2018). Further, the ability of myelin to restrict axonal sprouting may also enhance the selection of myelinated axons by reducing competition for downstream targets (McGee et al., 2005). Ablating myelin during the first 2 weeks of life not only reduces axonal diameter in the corpus callosum, but significantly reduces the heterogeneity in axonal diameter size, consistent with the notion that it promotes the selection of functional circuits (Jalabi et al., 2005). Network properties such as increased global efficiency and a reduction in small-worldness are markers of normal neurodevelopment (Huang et al., 2015) and improved cognition (Bullmore and Sporns, 2009; Huang et al., 2015; Bassett and Bullmore, 2017; Farahani et al., 2019) and are tightly linked with white matter development (Hagmann et al., 2010). Abnormal myelination may therefore account for the reduced global efficiency and increase in small-worldness seen in humans and mice exposed to EA (Ohashi et al., 2019; White et al., 2020). Although appealing and supported by indirect evidence additional work is needed to substantiate and clarify the role that myelination plays in selecting functional circuits during development (Figure 5).

One remarkable feature of myelin maturation in adult rodents is the presence of large number of unmyelinated axons accompanied by a sizable pool of OPC in many brain regions (Mitew et al., 2018; Steadman et al., 2020). This unique feature provides an important source of plasticity in adulthood that appears to be impaired by some forms of EA (Yang et al., 2017; Teissier et al., 2020). At the same time, agents that promote *de novo* myelination during development or even later in life may be used to compensate for earlier myelination and behavioral deficits caused by EA. Although significant *de novo* myelination has been seen in adult humans, it seems to be driven mainly by expansion of myelin in mature OL rather than a recruitment of OPC (Yeung et al., 2014). This difference in adaptive myelination may have important implications for challenges in translating successful interventions in adult rodents to humans and will benefit from additional work in non-human primate models of EA (see more on this issue below).

## EA Causes White-Matter Abnormalities in Humans, Non-Human Primates and Rodents

### Human Studies

Reduced corpus callosum size is one of the most consistent findings reported in individuals exposed to EA (Teicher et al., 1997, 2004; De Bellis et al., 1999, 2002; Cohen et al., 2006; Kitayama et al., 2007; Rusch et al., 2007; Andersen et al., 2008). These findings were seen in children and adults indicating that EA impairs normal myelin development in a manner that persists into adulthood. More recent work with diffusion MRI (dMRI) has extended the volumetric assessment to identify reduced fractional anisotropy (**FA**) – a measure of water diffusion and microstructural changes – in the corpus callosum of maltreated individuals (Jackowski et al., 2008; Paul et al., 2008; Teicher et al., 2010; Frodl et al., 2012; Huang et al., 2012; Lu et al., 2013; Bick et al., 2015; Rinne-Albers et al., 2016; McCarthy-Jones et al., 2018). Alterations in FA were also reported in other white matter tracks including the cingulum bundle, corona radiata, superior longitudinal fasciculus, inferior frontal-occipital fasciculus, fornix and the uncinate fasciculus, but these findings have been less reproducible (see Table 1). Bick and colleagues found that adopting children that were institutionalized during the first two years of life into supportive families was able to reverse some but not all of the white matter abnormalities at ages 8–11 (Bick et al., 2015). Interestingly, reduced FA in the corpus callosum persisted even after adoption suggesting that these changes might require longer time to correct or may not be fully correctable later in life (Table 1).

Using postmortem analysis, Tanti et al. reported increased density of mature oligodendrocytes in the ventro-medial prefrontal cortex of individuals with a history of EA and depression who died by suicide (DS-EA) compared to individuals with a history of depression who died by suicide but had no history of EA (DS) or a control group. The functional implications of this increase in the number of mature oligodendrocytes are yet to be clarified given that other markers of myelination (e.g., PLP, CNP, MAG, MOG, MOBP) were unaffected by or even decreased (e.g., MBP) in both DS-EA and DS groups compared to the control groups (Tanti et al., 2018), see also Figure 4. Moreover, as reviewed above, there is currently limited evidence for microstructural alterations in white matter prefrontal cortex in individuals with a history of EA.

### Clinical Significance

The corpus callosum is the largest white matter fiber track in the brain connecting the left and the right hemispheres and is responsible for the integration of sensory and motor information, executive function, language acquisition, and the management of emotional and social responses (Fryer et al., 2008; De Leon Reyes et al., 2020). The corpus callosum is organized in a homotypic manner in which equivalent areas in both hemispheres are connected. This organization ensures a specific flow of information across the caudal-rostral axis with sensory information located at the most caudal regions and prefrontal cortex executive flow of information across the rostral end (Figure 3A). Transcallosal projections undergo significant activity-dependent pruning during postnatal development and are therefore highly sensitive to environmental changes including EA (De Leon Reyes et al., 2020), see neuron 4 in Figure 5. Myelination of transcallosal projections starts at the caudal end reflecting early maturation of somatosensory and language capabilities early in childhood and more complex social and executive functions in late adolescence early adulthood (Bechler et al., 2018; De Leon Reyes et al., 2020). Additional work is needed to clarify whether excessive pruning of transcallosal projections is responsible for the corpus callosum abnormalities seen in individuals exposed to EA and whether abnormal myelination is actively contributing to this process. Addressing these questions will have important implications for the feasibility of reversing EA-mediated myelin abnormalities in the corpus callosum.

Alterations in CC and other white matter tracks are commonly seen in a broad range of psychiatric disorders (Fields, 2008; McCarthy-Jones et al., 2018; De Leon Reyes et al., 2020), many of which are likely to be attributed to EA (Teicher and Samson, 2016). The clinical significance of these abnormalities is a critical question that is yet to be clarified, especially given that FA changes are also seen in asymptomatic adult individuals with a history of EA (McCarthy-Jones et al., 2018). Moreover, some individuals with a complete absence of corpus callosum show little clinical deficits while others show very significant motor, social, and cognitive deficits (De Leon Reyes et al., 2020). Addressing the functional significance of these white matter abnormalities in humans is difficult, if not impossible, but important insights can be gained from work in animals.

### Non-human Primates

Reduced corpus callosum size and FA have also been reported in non-human primates exposed to EA (Sanchez et al., 1998; Coe et al., 2002; Jackowski et al., 2011; Howell et al., 2013), and Table 1. For example, 1 year old rhesus monkeys (*Macaca mulatta*) that were singly caged from 2 to 6 months and then group-housed for 6 months or maintained isolated for the entire 10 months (i.e., 2–12 months) showed reduced corpus callosum volume that was most pronounced in posterior regions. Reduced white matter volumes were also seen in the prefrontal cortex and parietal cortex and were associated with impaired acquisition of the delayed-non-matching to sample task (Sanchez et al., 1998). No differences were seen in corpus callosum size between animals that were group-housed during the last 6 months and those that were isolated for the entire 10 months (Sanchez et al., 1998). These findings are consistent with the adoption findings reported by Bick et al. (2015) and the notion that early deprivation causes long-term changes in corpus callosum size that are difficult to reverse. It will be interesting to test whether myelinating agents such as Clemastine fumarate can reverse some of the myelin and cognitive deficits seen in singly caged monkeys. This work will also clarify whether simply augmenting myelination can correct cognitive and behavioral abnormalities even in the absence of appropriate social stimulation.

Reduced corpus callosum size was also reported in 5 year old Bonnet monkeys (Macaca radiata) exposed to variable foraging demands between the ages of 2–6 months (Jackowski et al., 2011). This paradigm exposes mother-infant dyads to periods of time in which food availability is uncertain leading to increased maternal stress, insecure attachment, and increased emotional liability in the offspring (Andrews and Rosenblum, 1991). Finally, exposure to maternal abuse early in life was associated with reduced FA in the corpus callosum of adolescent rhesus monkeys that was correlated with elevated corticosterone levels measured at the peak of the abusive behavior when the infants were 1 month old (Howell et al., 2013).

In summary, non-human primates exposed to different types of EA show similar reduction in corpus callosum size and FA to those reported in humans (Table 1). Additional work is needed to clarify whether changes in corpus callosum size are primarily due to reduced number of callosal projections and whether abnormal myelination is simply a consequence of this reduction or a critical factor in destabilizing and eliminating these projections during a critical period of development. Work with non-human primates can also clarify whether myelinating agents can reverse some of the myelin abnormalities and cognitive deficits associated with EA and determine the age in which the administration of these agents is most efficacious (Figure 5). Work with non-human primate models of EA is crucial for addressing questions about timing for interventions because of important differences in the mechanisms responsible for adult *de novo* myelination in rodents and humans (Yeung et al., 2014).

### Studies in Rodents

Recent work in rodents provides some important insights into possible mechanisms by which EA alters myelination and complex behavior (summarized in Table 1). For example, using PLP-eGFP mice, Makinodan and colleagues found that isolating juvenile mice after weaning (P21-35) caused abnormal maturation of oligodendrocytes in the prefrontal cortex (**PFC**). Reduced activation of the Erbb3 receptor on oligodendrocytes, most likely due to reduced levels of its axonal ligand NRG1, were responsible for the abnormal myelination in the PFC and the impaired performance in the non-matching to place task. The use of PLP1-EGFP mice is an important technical detail because it revealed reduced ramification of myelin containing processes without affecting the density of these cells (Makinodan et al., 2012). Interestingly, abnormal myelination and behavioral deficits were seen when mice were socially isolated between P21-35, but not after P35. Group housing mice that were singly housed during the juvenile period with other socially isolated mice later in life did not reverse the myelin or behavioral deficits and similar behavioral and myelin deficits were seen in mice in which the Erbb3 receptor was eliminated from oligodendrocytes during the juvenile period (Makinodan et al., 2012). Interestingly, both the myelin and the behavioral deficits were reversed if previously isolated mice were later group-housed with control mice that were never isolated (Makinodan et al., 2017). Together these findings suggest that adequate levels of social contact during the juvenile period is necessary for Erbb3 mediated myelination and normal PFC function and that appropriate social milieu in adulthood can correct earlier deficits. It is currently unclear if the reduction in NRG1 levels seen in socially isolated mice is due to social deprivation and/or stress, why Erbb3 is no longer necessary for myelin formation after the sensitive period ended, and what aspects of social contact later in life are necessary to restore normal myelination and to improve PFC function later in life.

Yang et al. (2017) have shown that 3 h maternal separation (**MS**) during the first 3 weeks of life reduces myelination in the PFC in rats. These deficits were seen after weaning (P21) and in adult animals (P60) and were associated with reduced levels of mature oligodendrocytes and increased levels of OPC suggesting that MS impaired *de novo* myelination in adulthood. The blockade in OPC maturation was associated with reduced levels of the histone deacetylases 1 and 2 (HDAC1/2), and increased expression of WNT-related genes that blocked OPC differentiation (e.g., β-catenin, ID2, ID3, see also Figure 4). Administration of the HDAC1/2 inhibitor valproic acid (VPA) from P2-23 caused similar deficits in myelination and behavioral abnormalities seen in rats exposed to MS, whereas injecting the WNT antagonist XAV939 during the first 3 weeks of life reversed the myelination and behavioral abnormalities seen in MS. Together, these findings suggest that MS causes long-term increase in WNT signaling that blocks OPC differentiation and *de novo* myelination in the adult PFC (Figure 2), providing one of the most compelling mechanistic studies looking at this question in rodents. Importantly, this work is the first to demonstrate that myelinating agents, such as XAV939, can reverse the myelination and behavioral deficits seen in rat model of EA. An important limitation of this work is that it was done using cell lysates from the PFC and provide limited evidence for changes in gene expression in OPC. Moreover, MS did not impair myelination in the corpus callosum which is one of the most robust findings in humans and non-human primates (Table 1).

Teissier et al. (2020) found that mice exposed to 3 h split litter MS (**slMS**) during the first 2 weeks caused precocious maturation of OPC in the PFC at P21 and reduced number of OPC in the adult PFC of mice exposed to slMS. Precocious maturation of OPC early in life was associated with reduced neuronal activation in the developing PFC of slMS pups. Using chemogenetic activating and inhibitory viruses expressed in the PFC of newborn pups, the authors showed that inhibiting neuronal activity during the first two weeks of life led to precocious myelination in P15 pups, reduced OPC cell number in adulthood and caused similar behavioral deficits to those seen in mice exposed to slMS. Further, activating PFC neurons from P2-14 led to reduced myelination at P15 and exuberant myelination in adulthood that was able to reverse the behavioral deficits seen in slMS mice. The authors proposed that MS suppresses neuronal activation in the developing PFC leading to precocious maturation of OPC and depletion of OPC in adulthood (Teissier et al., 2020). Additional work is needed to clarify whether this reduction in OPC levels impairs adaptive myelination and its contribution to cognitive performance in adulthood. Although the work nicely demonstrates that neuronal inactivation causes similar alterations in myelin and behavioral changes to those seen in maternally separated mice, it is unclear whether the changes in myelination or other non-myelin related processes such as synaptic strength/pruning are responsible for the behavioral outcomes. Second, these findings seem inconsistent with a large body of work showing that OPC proliferation and differentiation increase with neuronal activation rather than neuronal inactivity (Stevens et al., 2002; Gibson et al., 2014; Hines et al., 2015; Mitew et al., 2018; Pan et al., 2020), although this might be a unique feature of the developing PFC and reflect underlying heterogeneity and region specific patterns of myelination discussed above (Figure 3B).

Work from several groups has shown that corpus callosum size is highly sensitive to early manipulations including early life stress in the mouse. Using handling, as a model of early stimulation, the Denenberg lab showed increased corpus callosum size in P110 adult rats exposed to handling during the first three weeks of life (Table 1). Increase in corpus callosum size was seen in males, while female rats showed reduced corpus callosum size and these rearing effects were no longer present in P225 rats (Berrebi et al., 1988). Handling is considered a form of early enrichment that is due to enhanced sensory stimulation induced by touching the pups and exposing them briefly to novel environmental milieu (Reeb-Sutherland and Tang, 2012). In addition, brief removal of the pups causes an increase in maternal care when the pups are reunited with the dam which is known to be a potent stimulator many aspects of neurodevelopment (Kaffman and Meaney, 2007; Couto-Pereira et al., 2016). This work is consistent with the notion that callosal projections and corpus callosum size are highly sensitive to sensory input early in life (Figure 5).

Duque et al. (2012) exposed mice pups to prolong daily maternal separation (4–8 h) from P2-P17, followed by early weaning at P17, a procedure they named maternal separation with early weaning (***MSEW***). Using this approach, they found a significant reduction in both gray and white matter that were more pronounced in the left hemisphere. They further confirmed their stereological and histological findings using dMRI showing reduced FA in the rostral and medial sections of the corpus callosum (Carlyle et al., 2012; Duque et al., 2012). Reduced myelination in the corpus callosum was associated with increased density of Olig2-positive cells (a marker of both OPC and OL, Figure 4) but reduced expression of genes expressed in mature OL suggesting that MSEM blocked OPC differentiation in the corpus callosum (Bordner et al., 2011; Duque et al., 2012). These findings are the first example showing reduced CC size in a rodent model of ELS. The translational utility of these changes is yet to be clarified because they exclusively affect the left hemisphere (Duque et al., 2012), a finding that has not been reported in humans, non-human primates or other mouse models. For example, using unbiased whole brain voxel analysis, White et al. (2020) found reduced volume and FA in the rostral regions of the corpus callosum in mice exposed to complex early life stress (White et al., 2020). These changes were seen in both males and females and were present in both the left and the right hemispheres. These findings are the first to use human imaging tools to confirm reduction is corpus callosum size and FA in a mouse model of ELS using unbiased whole-brain voxel analysis with rigorous correction for multiple comparisons (Table 1).

In summary, different paradigms of EA models in mice have been associated with abnormal myelination in adulthood. Most of the work in mice has focused on myelination in the PFC, but reduced corpus callosum size and FA have also been reported in some models. The available mechanistic studies suggest activation of WNT/β-catenin signaling impairs OPC differentiation in the adult PFC (Yang et al., 2017) while other forms of EA suppress neuronal activation in the developing PFC leading to precocious OPC maturation and OPC depletion later in life (Teissier et al., 2020). Importantly, emerging evidence suggests that myelinating agents can reverse the white matter and behavioral abnormalities seen in mice exposed EA or prolonged stress in adulthood (Liu et al., 2016; Yang et al., 2017).

## Conclusion and Future Directions

EA impairs white matter development in a manner that persists later in life in humans, non-human primates and rodents with reduced corpus callosum size and FA as the most consistent findings. Clarifying the mechanisms by which different forms of EA cause long-term changes in corpus callosum development and its impact on brain lateralization and behavior are important questions for translational research. Unfortunately, most of the work in rodents has focused on myelin development in the PFC with relatively few models showing abnormal corpus callosum development. Conversely, the effects of different forms of EA on myelination in the PFC of humans has received little attention and is worth revisiting.

Alterations in neuronal activity in response to deprivation or threat early in life are likely to play an important role in modifying myelin development (Figure 5). It is unclear however, whether neuronal activation increases or decreases myelin development. The emerging answer suggests that this might be circuit specific, begging the question of what signals mediate these different responses. Additional work is needed to clarify the role that compacted and uncompacted myelin plays in stabilizing axonal projections and synaptic strengthening during development. The distinction between myelin contribution to conduction velocity and metabolic coupling has not received enough attention with regard to their impact on circuit development and their functional consequences later in life. Differences in the involvement of compacted vs. uncompacted myelin may also explain why seemingly similar alterations in white matter are associated with very different functional consequences (De Leon Reyes et al., 2020).

Additional studies are needed to clarify the mechanisms by which EA reprograms OPC function later in life and how these changes impact adaptive myelination and behavior later in life. Finally, the availability of large number of myelinating agents (Murphy and Franklin, 2017) and genetic tools to block adaptive myelination (Makinodan et al., 2012; Pan et al., 2020) enable the field to test whether agents that increase myelination can reverse the myelination and cognitive deficits seen in animals exposed to EA, which circuits are more responsive, and when is the best time to apply them. Promising findings in rodents and especially in non-human primates will likely pave the way for new highly needed clinical interventions in humans.

## Author Contributions

RI reviewed the literature, wrote, and edited the manuscript. AK conceptualized the content, reviewed the literature, wrote, and edited the manuscript. All authors contributed to the article and approved the submitted version.

## Conflict of Interest

The authors declare that the research was conducted in the absence of any commercial or financial relationships that could be construed as a potential conflict of interest.
